# Computed tomography for the detection of distal radioulnar joint instability: normal variation and reliability of four CT scoring systems in 46 patients

**DOI:** 10.1007/s00256-016-2455-y

**Published:** 2016-08-24

**Authors:** Mathieu Wijffels, Wouter Stomp, Pieta Krijnen, Monique Reijnierse, Inger Schipper

**Affiliations:** 1 Department of Surgery-Trauma Surgery, Leiden University Medical Center, P.O. Box 9600, 2300 RC Leiden, The Netherlands; 2 Department of Radiology, Leiden University Medical Center, P.O. Box 9600, 2300 RC Leiden, The Netherlands

**Keywords:** Distal radioulnar joint, Instability, Normal values, CT scan, Distal radius fracture

## Abstract

**Objectives:**

The diagnosis of distal radioulnar joint (DRUJ) instability is clinically challenging. Computed tomography (CT) may aid in the diagnosis, but the reliability and normal variation for DRUJ translation on CT have not been established in detail. The aim of this study was to evaluate inter- and intraobserver agreement and normal ranges of CT scoring methods for determination of DRUJ translation in both posttraumatic and uninjured wrists.

**Materials and methods:**

Patients with a conservatively treated, unilateral distal radius fracture were included. CT scans of both wrists were evaluated independently, by two readers using the radioulnar line method, subluxation ratio method, epicenter method and radioulnar ratio method. The inter- and intraobserver agreement was assessed and normal values were determined based on the uninjured wrists.

**Results:**

Ninety-two wrist CTs (mean age: 56.5 years, SD: 17.0, mean follow-up 4.2 years, SD: 0.5) were evaluated. Interobserver agreement was best for the epicenter method [ICC = 0.73, 95 % confidence interval (CI) 0.65–0.79]. Intraobserver agreement was almost perfect for the radioulnar line method (ICC = 0.82, 95 % CI 0.77–0.87). Each method showed a wide normal range for normal DRUJ translation. Normal range for the epicenter method is −0.35 to −0.06 in pronation and −0.11 to 0.19 in supination.

**Conclusion:**

DRUJ translation on CT in pro- and supination can be reliably evaluated in both normal and posttraumatic wrists, however with large normal variation. The epicenter method seems the most reliable. Scanning of both wrists might be helpful to prevent the radiological overdiagnosis of instability.

## Introduction

Distal radius fractures comprise one in six fractures that are diagnosed at the emergency department [1–3]. The incidence of distal radial ulnar joint (DRUJ) instability after distal radius fractures varies from 0 to 35 % 1 year after trauma [4–7]. The complex bio-dynamics of the wrist show that during pronosupination the radioulnar fibers collaborate in preventing the ulna from luxating out of the sigmoid notch [8]. In extreme positions, additional stabilizing structures, such as the joint capsule, support the DRUJ from dislocation [9]. Posttraumatic changes may influence these stabilizing structures. Complete triangular fibrocartilage complex (TFCC) tears have been found to relate to DRUJ instability [4, 7]. Complete post-traumatic TFCC tears have been classified by Palmer as Palmer type 1B, C and D [10]. Although the stabilizing role the TFCC is important, other stabilizing structures can overcome DRUJ instability in case of complete TFCC tears [11–13]. Especially in Palmer type 1B the role of the disrupted TFCC seems not to influence DRUJ stability [6, 7, 14].

Diagnosing DRUJ instability clinically remains a challenge since the generally accepted clinical test for this condition, i.e., the stress-test, suffers from subjectivity and lack of validity and is a static test to evaluate a dynamic process [15]. Radiographs can be of additional value, although obtaining true lateral views is difficult and radiographs do not depict the dynamic process of DRUJ movement [4, 16–19]. Computed tomography (CT) of both wrists in pronation and supination may overcome these limitations [18, 20, 21].

Several methods for determining DRUJ translation on a wrist CT have been proposed [16, 19, 22–24]. To our knowledge only one paper has described the reliability of determining DRUJ instability during pro- and supination on CT scans [25]. Park et al. reported four scoring methods that were evaluated by three observers in the wrists of 45 healthy volunteers. They favored the subluxation ratio based on its reliability and simplicity, but reported substantial variation in normal values. Due to posttraumatic anatomical changes, the reliability of DRU translation measurement methods may differ from what Park et al. found, since they included healthy wrists only. Furthermore, findings may differ when the DRUJ stabilizing structures are stressed at maximal forearm rotation. The aim of our study was to determine the most reliable scoring method in terms of inter- and intraobserver agreement, to compare the reliability of measurements in injured and uninjured wrists and to determine normal ranges of radioulnar translation for these scoring methods in the patient population treated in our institution.

## Materials and methods

### Patients

All patients, over 18 years of age at trauma, treated conservatively for a distal radius fracture between May 2008 and February 2010 in our hospital, were eligible for inclusion in this prospective observational study. Patients were excluded if they (1) were unwilling or unable to provide informed consent, (2) had systemic diseases such as rheumatoid arthritis and systemic lupus erythematosus (SLE) or (3) had contralateral wrist injury. Eligible patients received an invitation letter for a study visit. Informed consent was obtained from all individual participants included in the study. The institutional medical ethics review board approved the study.

### Study procedure

After informed consent had been obtained, the presence of pain in free forearm rotation was documented using a visual analog scale. This was a 100-point scale, ranging from 0 (no pain) to 100 (worst pain imaginable). Radiological DRUJ translation was assessed using CT. Pro- and supination was measured using a goniometer and expressed as absolute range and as a percentage of the non-injured wrist.

The fractures were classified based on the baseline radiograph by one reader (MW) according to the Comprehensive Classification of Fractures in type A (extraarticular), B (partial intraarticular) and C (complete intraarticular) fractures [26]. No control radiographs were taken at final follow-up; fracture healing was determined on the CT with reformatting.

### Computed tomography

The CT scans (Aquilion One or 64, Toshiba, Tokyo, Japan) were made in prone position with both arms above the head and extended elbows, in both maximal pronation and maximal supination (tube voltage 120 KV, tube current 70 mA, rotation time 0.5 s, slice thickness 0.5 mm, slice increment 0.4 mm). Patients were verbally instructed to maximize forearm pro- and supination during CT scanning.

The scanned area covered 5 cm proximal of the radiocarpal joint to 1 cm distal of the metacarpal heads. Post-processing was performed by trained radiology employees and included 2-mm coronal and sagittal reformats as well as 2-mm axial reformats perpendicular to the axis of the styloid process and ulnar shaft. Each wrist was reformatted separately and on a different slide.

### Radiological assessment of DRUJ instability

DRUJ translation was quantified using four methods: the radioulnar line method [16, 23], subluxation ratio method [25], epicenter method [24] and the radioulnar ratio method [22]. All methods measure radioulnar translation by evaluating the ulnar position relative to the radius resulting in a ratio (see below for a detailed description). Prior to the evaluation of the study CT scans, the two observers (MW, 6 years of experience in traumasurgery, and WS, 3 years of experience in radiology) scored ten CT scans of wrists not involved in this study according to the four methods for training purposes.

The axial reformatted CT image of each wrist showing the largest area of the sigmoid notch, including the Lister tubercle and ulnar head, were selected by each individual observer for measurement of ulnar translation in both pro- and supination. All CT images were independently assessed in random order by both observers, who were blinded to patient and clinical characteristics. At a minimum of 3 weeks after the first series of reviews, one observer (MW) assessed all CT slides for a second time in random order for the determination of intraobserver reliability.

Quantification of the ulnar position relative to the radius of both the injured and uninjured wrist was done in four ways:

1. According to the radioulnar line method [16, 23], two lines are drawn: one through the volar ulnar and radial borders of the radius (Fig. 1, line a) and a second through the dorsal ulnar and radial borders of the radius (line b). The maximum distance of the ulnar head outside these two lines is measured, perpendicular to line a, line CD. A fourth line connecting the two edges of the sigmoid notch is drawn, which defines the length of the sigmoid notch (length AB). The ratio of CD to AB is calculated. Volar dislocation of the ulnar head relative to the radius is considered negative, dorsal dislocation as positive. If the ulnar head is situated between line A and B, the value is recorded as 0.

**Fig. 1:**
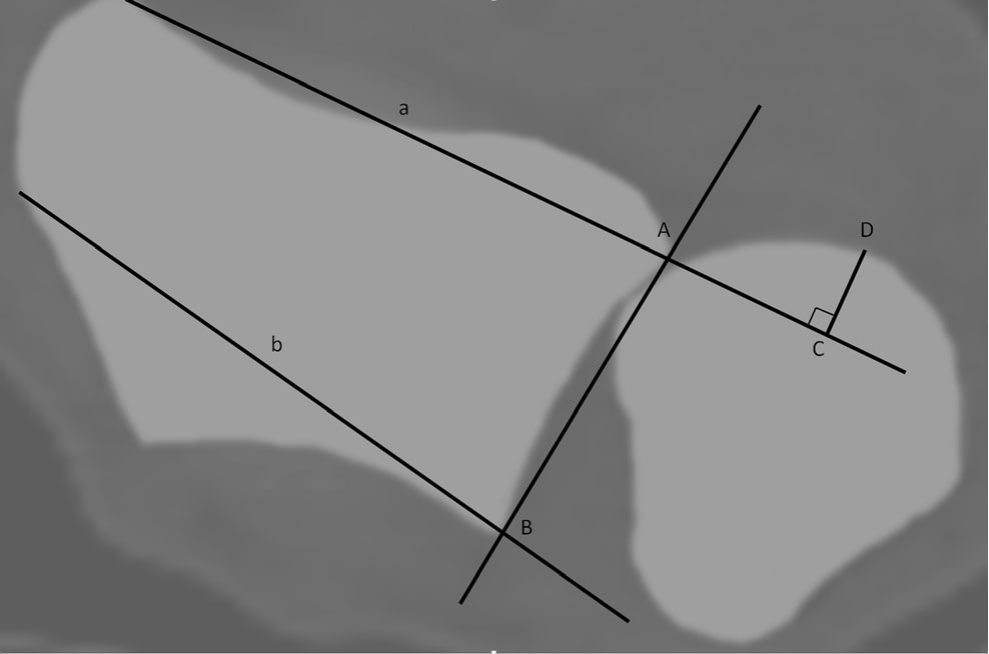
The radioulnar line method = CD/AB: the amount of ulnar head volar or dorsal from the *ulnar line* is measured (CD). The ratio of this length to the length of the sigmoid notch (AB) is calculated. Wrist in supination
2. According to the subluxation ratio method [25], a line connecting the two edges of the sigmoid notch (point A and B) is drawn, which defines the length of the sigmoid notch (Fig. 2, length AB). Two lines (line a and line b) are drawn perpendicular to this line and cross the edges of the sigmoid notch. The maximum distance of the ulnar head outside line a or b is measured perpendicular to this line (distance CD). The ratio between the length of extraarticular ulnar head and the sigmoid notch length is calculated (CD/ AB). Volar dislocation of the ulnar head relative to the radius is considered negative, dorsal dislocation as positive.

**Fig. 2:**
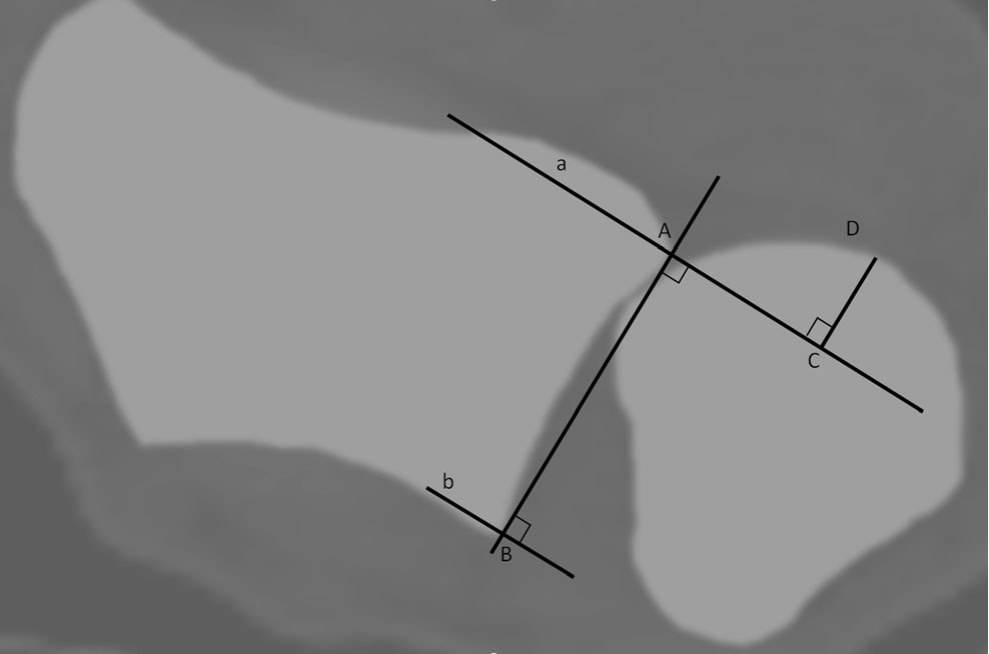
The subluxation ratio method = CD/AB. The length of the sigmoid notch is defined by length AB. The distance of the ulnar head outside line a or b is measured perpendicular to this line (distance CD). The ratio between the length of the extraarticular ulnar head and sigmoid notch length is calculated (CD/ AB). Wrist in supination
3. According to the epicenter method [24], a line connecting the two edges of the sigmoid notch is drawn (Fig. 3, line AB), which defines the length of the sigmoid notch. Using two circles the center of the ulnar head and ulnar styloid process is marked point a and b, respectively. The center of rotation of the DRUJ is marked by point D, the crossing of a perpendicular to line AB through point c, halfway on the line connecting point a and b. The distance between point D and the midpoint of the sigmoid notch, point C, is measured. The ratio between distance CD and AB is calculated. Volar dislocation of the ulnar head relative to the radius is considered negative, dorsal dislocation as positive.

**Fig. 3:**
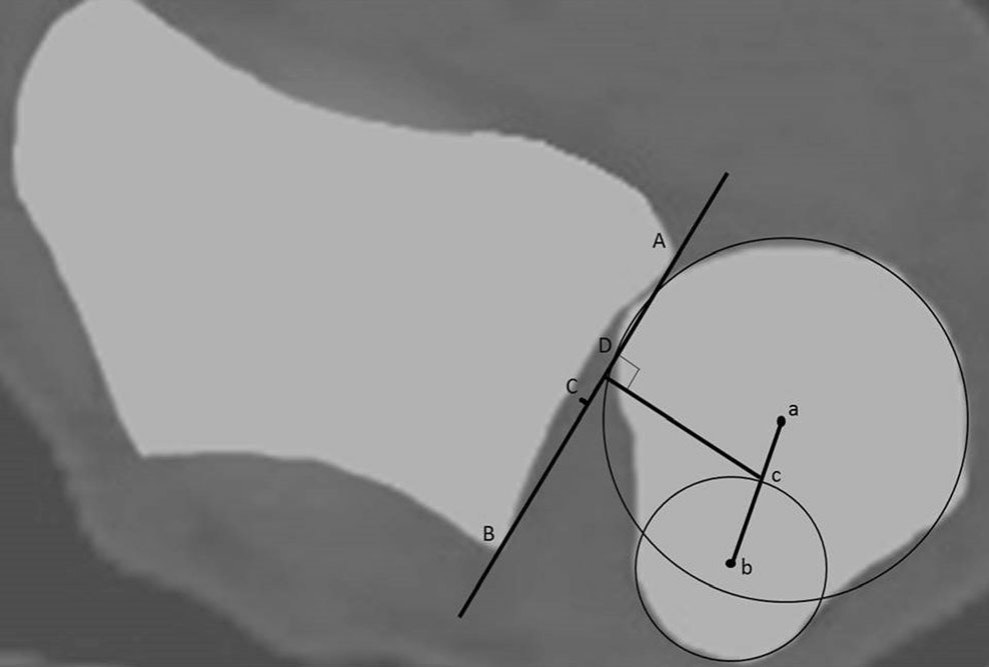
The epicenter method = CD/AB. The centre of rotation (point D) is defined by the center of the ulnar head and ulnar styloid process (point a and b, respectively). Point c is half the length of line ab. The crossing of the *line*, perpendicular to line AB and through point c, defines point D. The distance between point D and the midpoint of the sigmoid notch, point C, is measured. Wrist in supination
4. According to the radioulnar ratio method [22], a line (Fig. 4, line AB) connecting the two edges of the sigmoid notch is drawn, which defines the length of the sigmoid notch. A second line (line C) is drawn, perpendicular to the first one and through the center of the ulnar head (point C), defined by a circle facing the articular surface. The ratio between the distance from the cross point of the two lines (point D) to the volar edge of the sigmoid notch (length AD) and the length of sigmoid notch (length AB) is calculated.

**Fig. 4:**
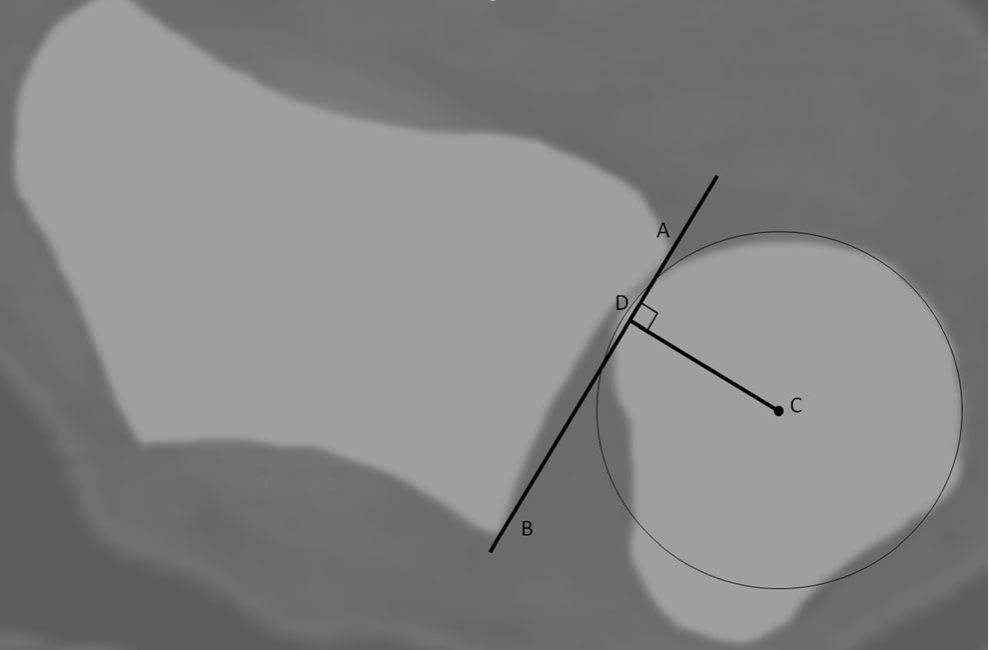
The radioulnar ratio method = AD/AB. The length of the sigmoid notch is measured (length AB). A line perpendicular to line AB and the center of the ulnar head defines point D. Wrist in supination

### Statistical analysis

To evaluate the reliability (inter- and intraobserver agreement) of DRUJ translation measurements on CT, intraclass correlation coefficients (ICCs) with their 95 % confidence interval (CI) were calculated using the two-way random model for absolute agreement. The ICCs were interpreted according to Landis and Koch who proposed that values 0.01 to 0.20 indicate slight agreement; 0.21 to 0.40, fair agreement; 0.41 to 0.60, moderate agreement; 0.61 to 0.80, substantial agreement; and 0.81 to 1, almost perfect agreement [27, 28]. Statistical difference in agreement was defined by absence of overlap of the 95 % confidence intervals of the ICCs.

Pro- and supination were compared between groups using the paired samples t-test.

The number of observations needed was calculated to ensure significant agreement if the agreement was at least 0.4, with an alpha of 0.05 and a beta of 0.2. The minimal sample size of wrist CT scans to be reviewed was found to be 87 [29].

The normal range of ulnar translation for each method was based on CT scans of the uninjured wrists and defined as the mean value ±2 SD for both pro- and supination.

## Results

One hundred fifty-eight patients met the inclusion criteria and were invited for a study visit. Thirty-six patients were lost to follow-up, and 73 were unwilling to participate. No patients were excluded based on a systemic disease or contralateral wrist injury. Three of the remaining 49 participants had an incomplete CT scan, leaving 46 participants with 92 wrist CTs (pro- and supination) for analysis. The baseline characteristics of the included patients are shown in Table 1. Four patients (2 with a complete intraarticular fracture type and 2 with an extraarticular fracture type) suffered from pain in the injured wrist with a VAS score of 40, 50, 1 and 60 points, respectively. No significant differences were found comparing pro- and supination between injured and non-injured wrists (*p* = 0.13 and *p* = 0.84, respectively). No differences in pro- and supination in the injured wrist (expressed as percentage of the non-injured wrist) were found comparing the patients with and without pain in free pronosupination (*p* = 0.78 and *p* = 0.06, respectively).

**Table 1:** Baseline characteristics of the 46 included patients

Sex (n)		
	Male	8
	Female	38
Mean age (years)		56.5 (SD 17, range 18–87)
Mean follow-up (years)		4.2 (SD 0.5, range 3.3–5.0)
Mean pronation^a^		
(Degrees)/% of healthy wrist		85.5 (SD 9.3)/98.1 (SD 9.0)
Mean supination^a^		
(Degrees)/% of healthy wrist		91.2 (SD 12.9)/97.5 (SD 25.7)
AO fracture type (n)		
	A	22
	B	4
	C	20

^a^Injured wrist

All distal radius fractures were consolidated at final follow-up.

The highest inter- and intraobserver agreement, independent of position or posttraumatic state of the wrist, was found for the epicenter method (ICC = 0.73 95 % CI 0.65–0.79 and ICC = 0.82, 95 % CI 0.77–0.87, respectively) as presented in Table 2. When wrist position was taken into account interobserver agreement remained best for the epicenter method in both pronation (ICC = 0.47, 95 % CI 0.16–0.67) and supination (ICC = 0.72, 95 % CI: 0.58–0.81). Accounting for wrist position, intraobserver agreement was best for the radioulnar line method in both pronation (ICC = 0.67, 95 % CI 0.53–0.77) and supination (ICC = 0.82, 95 % CI: 0.74–0.88). All ICCs were higher for supination measurements as compared to the corresponding pronation measurements (Further data not shown.)

**Table 2:** Intraclass correlation coefficients (ICCs) with 95 % confidence interval (CI) for inter- and intraobserver agreement on four scoring methods for measuring DRUJ instability on 184 CT scans of the wrist

Method	Interobserver agreement (ICC, 95 % CI)	Intraobserver agreement (ICC, 95 % CI)
Radioulnar line	0.53 (0.22–0.71)	0.75 (0.68–0.81)
Subluxation ratio	0.51 (0.20–0.69)	0.64 (0.54–0.72)
Epicenter	0.73 (0.65–0.79)	0.82 (0.77–0.87)
Radioulnar ratio	0.68 (0.55–0.76)	0.76 (0.70–0.82)

The ICCs for interobserver agreement of all four methods, separately for injured and uninjured wrists, are presented in Table 3. Agreement on measurements in supination with the epicenter method for the injured wrists was almost perfect (ICC = 0.82, 95 % CI 0.69–0.89) and was significantly better than that for the uninjured wrists.

**Table 3:** Intraclass correlation coefficients (ICCs) with 95 % confidence interval (CI) for interobserver agreement on four scoring methods for measuring DRUJ instability in pro- and supination on CT

Method	Pronation ICC (95 % CI)		Supination ICC (95 % CI)	
	Injured (*n* = 46)	Non-injured (*n* = 46)	Injured (*n* = 46)	Non-injured (*n* = 46)
Radioulnar line	0.16 (−0.07−0.40)	0.37 (0.02–0.62)	0.68 (0.32–0.84)	0.33 (0.05–0.56)
Subluxation ratio	0.20 (−0.06−0.45)	0.28 (−0.02−0.53)	0.62 (0.29–0.80)	0.22 (−0.04−0.46)
Epicenter	0.54 (0.19–0.75)	0.42 (0.08–0.66)	0.82 (0.69–0.89)	0.47 (0.18–0.68)
Radioulnar ratio	0.52 (−0.01−0.78)	0.30 (−0.10−0.62)	0.60 (0.37–0.76)	0.35 (0.07–0.58)

The ICCs for intraobserver agreement of all four methods, separately for injured and uninjured wrists, are presented in Table 4. The best and almost perfect agreement was found for the radioulnar line method on supination CT imaging of the injured wrist (ICC = 0.92, 95 % CI 0.86–0.95), which was significantly better compared to the uninjured wrist.

**Table 4:** Intraclass correlation coefficients (ICCs) with 95 % confidence interval (CI) for intraobserver agreement on four scoring methods for measuring DRUJ instability in pro- and supination on CT

Method	Pronation ICC (95 % CI)		Supination ICC (95 % CI)	
	Injured (*n* = 46)	Non-injured (*n* = 46)	Injured (*n* = 46)	Non-injured (*n* = 46)
Radioulnar line	0.54 (0.28–0.72)	0.74 (0.58–0.85)	0.92 (0.86–0.95)	0.62 (0.40–0.77)
Subluxation ratio	0.23 (−0.03−0.47)	0.62 (0.40–0.77)	0.90 (0.83–0.94)	0.49 (0.24–0.68)
Epicenter	0.55 (0.31–0.72)	0.60 (0.38–0.76)	0.84 (0.73–0.91)	0.45 (0.19–0.66)
Radioulnar ratio	0.61 (0.39–0.77)	0.65 (0.45–0.79)	0.63 (0.42–0.78)	0.64 (0.44–0.79)

In Table 5 the mean ratios of ulnar translation for all four scoring methods for injured and uninjured wrists in pro- and supination are presented. The normal range of ulnar translation ratios differed from 30 % in the epicenter method in supination to 59 % in the subluxation ratio in pronation.

**Table 5:** Mean ratio values with standard deviation (SD) of radioulnar deviation measured with the four scoring methods on CT for the injured and non-injured wrists and normal values based on the non-injured wrist in pro- and supination

Method	Injured wrist Mean (SD)		Non-injured wrist Mean (SD)		Normal range	
	Pronation	Supination	Pronation	Supination	Pronation	Supination
Radioulnar line	−0.05 (0.11)	−0.22 (0.18)	0.14 (0.15)	−0.15 (0.12)	−0.15–0.43	−0.39–0.08
Subluxation ratio	−0.02 (0.11)	−0.20 (0.17)	0.05 (0.15)	−0.17 (0.11)	−0.25–0.34	−0.39–0.04
Epicenter	−0.17 (0.09)	−0.01 (0.13)	−0.15 (0.10)	0.04 (0.08)	−0.35–0.06	−0.11–0.19
Radioulnar ratio	0.56 (0.11)	0.29 (0.16)	0.58 (0.09)	0.33 (0.12)	0.39–0.77	0.09–0.58

## Discussion

In this study the best interobserver agreement of four scoring methods for determination of DRUJ translation by means of CT scan was established using the epicenter method. This method also showed good corresponding intraobserver agreement values, and agreement was better for injured wrists compared to uninjured wrists. Based on these data the epicenter method seems the most reliable method to evaluate distal radioulnar translation on CT scans.

Our findings are in contrast with the data published earlier by Park et al. [25]. They found a substantial to almost perfect interobserver agreement for the radioulnar line method in supination and pronation, respectively, for uninjured wrists. A plausible explanation for the difference between the findings of Park and ours is hard to find. In both studies the CT protocol used was identical, and wrist positioning and image selection were performed concordantly. The inclusion of posttraumatic wrists in our study had no negative effect on the reproducibility of the measurements. Furthermore, Park et al. favored the subluxation ratio for its simplicity [25]. We did not evaluate the practical aspects of the various scoring methods. From personal communication between the observers it can be concluded that the radioulnar line method is the quickest and easiest.

Hess et al. presented a novel technique using ultrasound to evaluate DRUJ dislocation and found both the sensitivity and specificity to be over 80 % for complete TFCC lesions [30]. Although promising, it is hard to compare these results with our data since we did not analyze predictive values. Furthermore, Hess and colleagues did not evaluate agreement between observers.

### Normal ranges

During pronation of the wrist, the unstabilized ulnar head tends to move dorsal relative to the radius. Ulnar translation is therefore one of the indicators of insufficiency of the DRUJ stabilizers, i.e., DRUJ instability. The ratios calculated using the four methods were translated into a percentage representing the amount of ulnar head dislocation outside the sigmoid notch. Using the epicenter method, which had the best interobserver agreement, normal values of ulnar translation varied from 35 % volar dislocation to 6 % dorsal dislocation in pronation and from 11 % volar dislocation to 19 % dorsal dislocation in supination. Using the radioulnar line method, Mino described the position of the ulnar head within the lines through the dorsal and volar border in every rotational position [16, 23]. This resulted in a narrow window for normal values, which is smaller than what was found based on our data. Using Mino’s criteria would easily lead to high numbers of patients with uninjured wrist function who are considered to have an abnormal DRU joint and DRU instability (false-positive findings). On the other hand, Park et al. [25] reported normal values in uninjured wrists varying from 27 % volar to 35 % dorsal dislocation, a wider range than what we found. Based on Parks’ wide normal range, one may judge an actual unstable DRUJ as normal on the CT scan, leading to a false-negative outcome. These findings correspond with the results of Kim and colleagues who found a poor correlation between CT findings and clinical DRUJ assessment [31]. We therefore recommend to interpret normal values with caution when determining DRUJ instability on CT scans. To avoid false-positive and -negative findings, we suggest, in accordance with Nakamura and colleagues, to compare the healthy and injured wrists of a patient expected to have DRUJ instability [19]. The uninjured wrist will reflect the normal laxity of the DRUJ in both pro- and supination. However, no studies on this theory for any of the four scoring methods have been published [19]. For patients with injuries of both wrists, normal values as presented by Park, Mino and our data are the best available reference [23, 25].

**Fig. 5:**
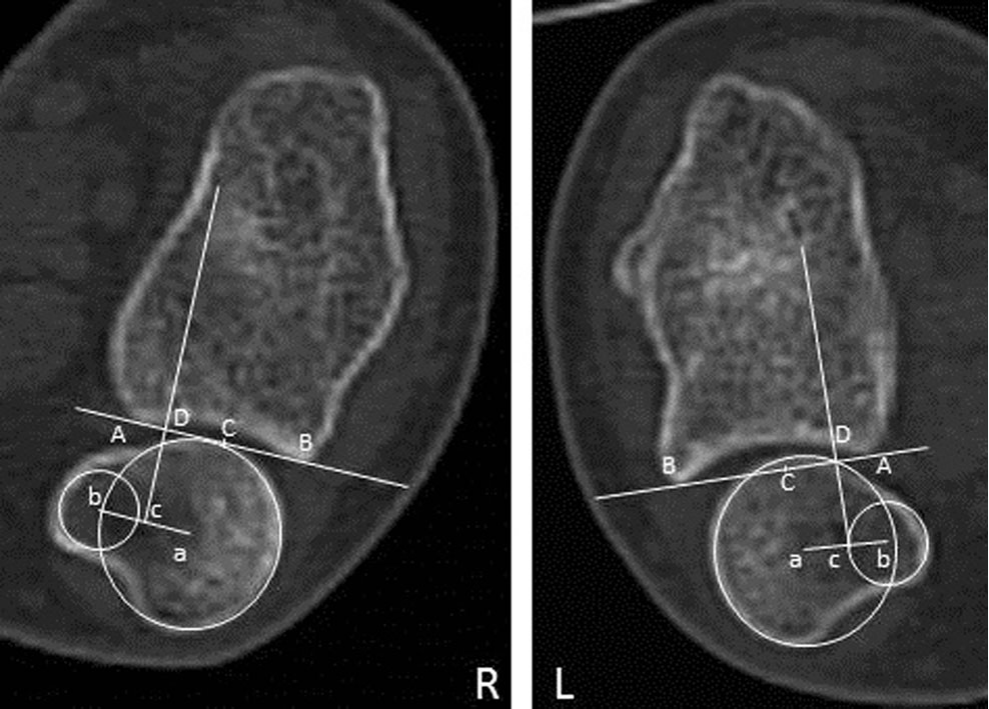
The CT scan in pronation of a 24-year-old female, 4 years and 4 months after a complete intraarticular fracture on the left side. Using the epicenter method, this wrist was measured out of the normal range, compared to normal values based on mean measurements. She indicated no (0) pain using the VAS score

This study had a number of limitations. Although the protocol stated that the largest area of the sigmoid notch should be selected, including Lister’s tubercle on the axial reformatted CT images, it seems probable that different slides were selected for conducting the measurements (Fig. 5). A computerized system may overcome this shortcoming. Another limitation of this study was that only the reliability of determination of clinical DRUJ instability on CT scans could be evaluated. Since no reliable and objective test is available for diagnosing DRUJ instability, we were not able to evaluate the validity of CT scans for determination of clinical DRUJ instability. Nevertheless, these results are valuable given the lack of reliable data on the evaluation of methods for diagnosing radiological DRUJ instability using CT in injured wrists.

## Conclusion

Measurements for DRUJ instability on CT in pro- and supination can be reliably performed in both normal and posttraumatic wrists. The epicenter method seems the most reliable method for scoring DRUJ translation using CT scan of the injured wrist. There is large normal variation in DRUJ translation. Scanning of both wrists might be helpful to prevent the radiological overdiagnosis of instability.
